# Impact of Daily Maximum Temperature on Emergency Department Arrivals and Acuity Levels

**DOI:** 10.5811/westjem.42263

**Published:** 2025-09-25

**Authors:** Catharina Giudice, Nicholas J. Arisco, Zilin Lu, Bryan Stenson, Caleb Dresser

**Affiliations:** * Beth Israel Deaconess Medical Center, Department of Emergency Medicine, Boston, Massachusetts; † Harvard T.H. Chan School of Public Health, Harvard Chan Center for Climate, Health and the Global Environment, Department of Environmental Health, Boston, Massachusetts; ‡ Harvard T.H. Chan School of Public Health Department of Global Health and Population, Boston, Massachusetts; § Tufts University, Department of Public Health and Community Medicine, Boston, Massachusetts

## Abstract

**Introduction:**

Heatwaves are becoming more frequent and severe globally. Heat is associated with increases in emergency department (ED) volumes and higher morbidity for a range of chronic conditions. We describe how temperature impacts ED arrivals at different acuity levels.

**Methods:**

We obtained time-series records for daily ED arrivals stratified by Emergency Severity Index (ESI) from 2010 – 2019 from hospital records. Wet-bulb temperature was the exposure of interest; analysis was controlled for precipitation, snow, wind speed, day of week, and federal holidays. We fitted a Poisson model for each ESI category and estimated the association between temperature and ED arrival acuity with a distributed lag non-linear model with three days of lag to account for delayed health effects of temperature.

**Results:**

We analyzed data for 3,652 days totaling 556,663 arrivals between 2010 – 2019. At lag 0, lower temperatures were associated with a reduced relative risk of arrival to the ED for ESI 2, ESI 3, and total arrivals. At higher temperatures, ESI 2 and ESI 3 showed an increased relative risk of arrival (wet-bulb exposure of 25°C at 0-day lag: ESI 2 RR = 1.06 [1.02–1.10]; ESI 3 RR = 1.04 [1.01–1.07]). While not statistically significant, ESI 1 exhibited a subtle increase in arrivals at the highest temperatures while ESI 4 & 5 displayed a subtle decrease in relative risk of arrivals under these conditions.

**Conclusion:**

Extremes of temperature, particularly heat, appear to affect ED arrivals differently across different acuity levels. Medium- to higher-acuity presentations appear to be more responsive to heat, with a statistically significant increase in ED presentations on days with the highest heat burden. The highest acuity presentations became numerically but not statistically more frequent on days with the highest heat burden, while the lowest acuity presentations decreased numerically but not statistically in these conditions.

## INTRODUCTION

Climate change is becoming an increasingly important determinant of health, with potential operational implications for acute care systems. The global annual average temperature for 2024 has already surpassed pre-industrial era levels by 1.6 °C^1^ and, under the most optimistic scenarios, are expected to exceed 2.7 °C during the 21st century.^2^ Episodes of extreme heat are becoming more frequent, prolonged, and extreme throughout the United States. By midcentury, heat indexes exceeding 37.8 °C are expected to triple in the Northeast under an intermediate greenhouse gas emission scenario.^3^ This has profound implications for health and healthcare delivery, including increases in all-cause mortality, healthcare utilization, rates of death from cardiovascular and respiratory diseases,^4^ mental health issues,^5^ accidental and non-accidental trauma,^6^ and adverse birth outcomes.^7^ Broadly, heat is known to be a significant environmental contributor to mortality.

Emergency departments (ED), as the primary access point for acute healthcare delivery, also face increases in volume and operational burden due to extreme heat.^8^ Previous studies have shown a relationship between higher temperatures and increased overall ED use.^8^,^9^ However, there is wide variation in the disease severity of patients presenting to EDs and the level of resources required to provide appropriate care. Few studies have looked at how temperature and other environmental variables affect the acuity level of emergency arrivals.^10^–^13^ At a facility level, understanding how heat impacts patient presentations across each of the five Emergency Severity Index (ESI) levels may facilitate more informed resource allocation and staffing decisions.

At a community level, knowledge of the range of pathologies for which patients seek care during extreme heat may inform policies and programs intended to preserve health and reduce burden on acute care systems. Our aim in this study was to evaluate relationships between ambient temperature and ED arrival volume across the spectrum of acuity at an urban hospital in the Northeastern US.

## METHODS

### Setting

This study was completed at an urban, academic medical center in the Northeast region of the US with an annual ED volume of approximately 55,000 patients per year. We chose the study period 2010 – 2019 to exclude the COVID-19 pandemic, during which ED visits did not follow typical volume patterns. The predominant climate in the study region is humid subtropical, characterized by hot, humid summers and an average snowfall of 50 inches during the winter months. Thunderstorms are common from June–August.^14^

### Data Sources

We obtained daily ED arrivals stratified by ESI from 2010–2019 from electronic health records (EHR) at a large, urban hospital. The ESI is a five-level scale used to assess patient stability, risk of deterioration, and anticipated resource needs.^15^ An ESI 1 is assigned to patients with severe medical conditions requiring immediate lifesaving interventions, while ESI 5 is assigned to patients who are stable and expected to require minimal resources for care. Due to the relatively low volume of patients in the ESI 4 and ESI 5 categories and their similar relative clinical stability and low resource needs, we combined these two groups in this analysis.

We obtained historic weather records from the National Oceanic and Atmospheric Administration, National Centers for Environmental Information. Boston Logan International Airport Station (WBAN:14739) was selected due to its proximity to the study site (8.5 km). Federal holidays occurring during the study period were obtained from government historical records.

**Population Health Research Capsule**

What do we already know about this issue?

*Heat is a significant environmental contributor to mortality and has been shown to increase overall emergency department (ED) utilization*.

What was the research question?

*Does the temperature responsiveness of ED arrivals differ by case acuity level?*

What was the major finding of the study?

*Heat (25 °C) increased ED arrivals for Emergency Severity Index (ESI) 2 (RR=1.06 [1.02–1.10]) and ESI 3 (RR=1.04 [1.01–1.07])*.

How does this improve population health?

*Understanding the impact of extreme heat on ED arrivals informs data-driven climate preparedness and may guide staffing and resource planning during heat waves*.

There is evidence suggesting equivalence between different temperature metrics used to assess heat exposure in population health research.^16^ For this study, we chose wet-bulb daily maximum temperature as the exposure index, as it combines both ambient temperature and humidity and may better correlate with physiological response to heat.

### Statistical Analysis

We performed a Poisson regression time series analysis using a framework of distributed lag nonlinear models (DLNM) to estimate the effect of temperature (°C) on ED arrival volumes for each ESI category. Model specification and choice of control variables were based on prior ED arrival predictive modeling studies.^17^ We included several control variables, including daily precipitation (millimeters [mm]), wind speed (meters per second), snow (mm), day of the week, day of holiday, day before holiday, and day after holiday. Our exposure of interest was wet-bulb daily maximum temperature, which was modeled using a cubic B-spline with 2 degrees of freedom to account for seasonality and long-term trends in accord with previous studies.^18^,^19^ To evaluate for a possible “lag” effect, which is to say, to evaluate for the possibility that the influence of temperature extremes are most pronounced on one of the days subsequent to the day of the temperature extreme, we evaluated the influences of lags of 0, 1, 2, and 3 days.^20^

Lags were modeled using a cubic B-spline with 4 degrees of freedom. Wind speed was controlled for the day of ED arrival. To evaluate the possibility that the influence of snow and precipitation are most pronounced on one of the days after these events, we evaluated the influence of lags of 0, 1, and 2 days. In the cross-basis function, three internal knots were placed at the 10th, 50th, and 90th percentiles for each environmental variable. We defined the median value of temperature (11.7°C) as the baseline temperature or centering value for calculating the relative risk (RR). We plotted the RR against temperature for each lag and for all lags aggregated.

We conducted sensitivity analysis to test the robustness of changing model choices for daily lagged models, using average and minimum temperature instead of maximum temperature as the primary exposure, and altering the exposure variable choice to include dry-bulb temperature and humidity (Appendix). We used Akaike information criteria values to help guide model selection. All analysis was performed with R software v4.3.2 (R Foundation for Statistical Computing, Vienna, Austria) using package DLNM (v2.4.7) for the distributed lag nonlinear model.

### Ethics

This study was approved by the Committee on Clinical Investigations at Beth Israel Deaconess Medical Center (institutional review board) Protocol 2022P000002).

## RESULTS

**Figure 1:**
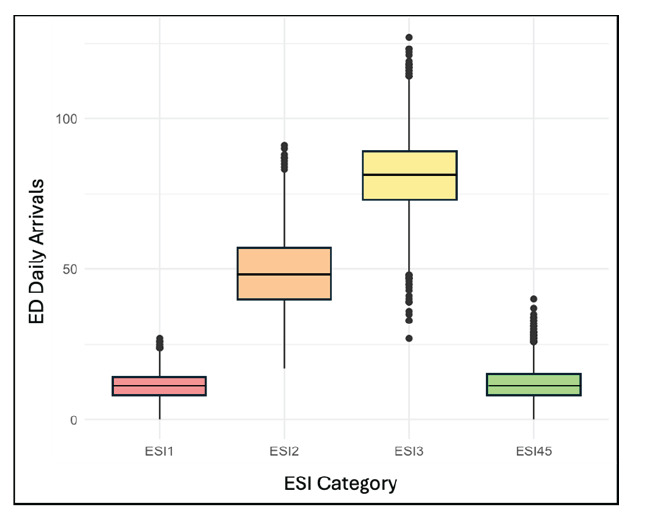
Box plot of total emergency department arrivals per day stratified by Emergency Severity Index category. *ED*, emergency department; *ESI*, Emergency Severity Index.

**Figure 2:**
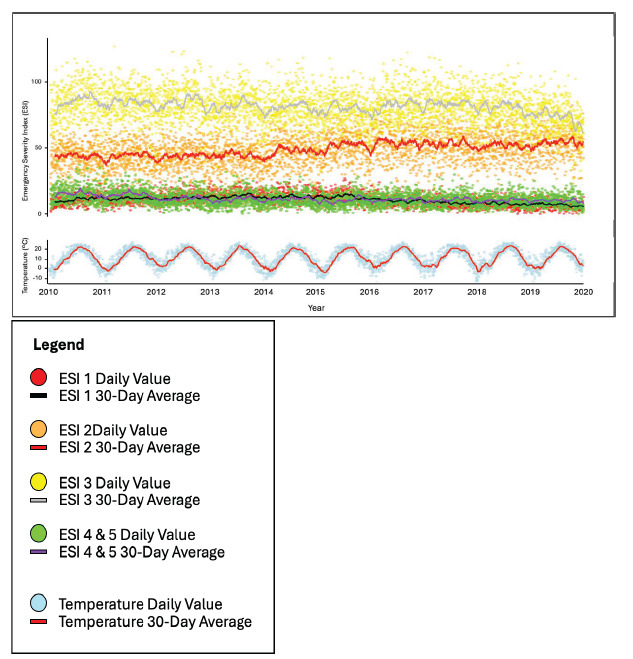
Daily time series of ED arrival for each Emergency Severity Index category and maximum wet-bulb temperature for the study period. *ED*, emergency department; *ESI*, Emergency Severity Index.

The dataset included 3,652 days and totaled 556,663 arrivals during the study period from 2010 – 2019. The ESI 3 group accounted for the greatest number of arrivals at 297,074 (53%), followed by ESI 2 at 178,031 (32%), ESI 4 and 5 combined at 42,086 (7.6%), and ESI 1 at 39,472 (7.1%) (Figure 1). When aggregated over the study period, total ED arrivals were highest on Mondays and in July, and lowest on Sundays and in February, aligning with established trends in ED volume. Daily time series stratified by ESI indicate a high degree of variability across all ESI groups, without obvious cyclical patterns (Figure 2). Daily maximum wet-bulb temperature for the study period ranged from −13.8 °C to 27.2 °C with a mean of 10.8 °C (SD 8.6 °C).

**Figure 3:**
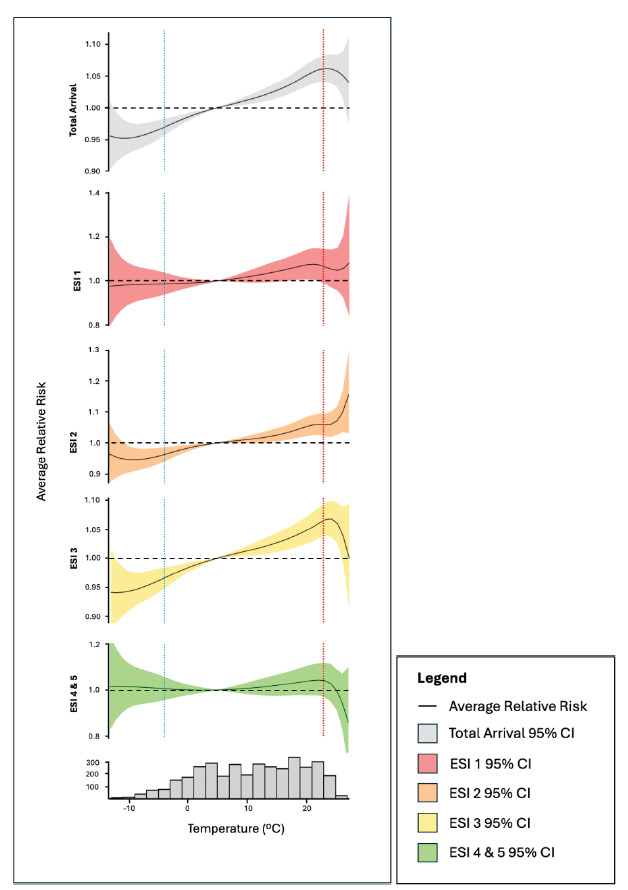
Relative risk (RR) of emergency department presentation in relation to daily maximum temperature at lag 0, stratified by Emergency Severity Index (ESI) category. Solid line is the exposure-response curve (best linear unbiased prediction) with 95% confidence interval (CI) shaded in light red (ESI 1), orange (ESI 2), yellow (ESI 3), and green (ESI 4 and 5). Horizontal dotted line represents RR = 1. Vertical dotted lines represent 5th percentile of temperature (blue = −3.9 °C) and 95th percentile of temperature (red = 23.3 °C).

Figure 3 shows the cumulative exposure-response curves for the different ESI categories with relative risk of 1 set at the median maximum temperature for the study period (11.7 °C). The temperature distribution highlights how, at the extremes of the temperature range, only a small number of hot or cold days occur. At lag 0, lower temperatures were associated with a reduced RR of arrival to the ED for ESI 2, ESI 3, and total arrivals. At higher temperatures, ESI 2, ESI 3, and total arrivals showed an increased RR of arrival. ESI 1 and ESI 4 & 5 did not demonstrate a statistically significant association with temperatures across most temperature ranges.

On the hottest 5% of days, there was a small numeric increase in the RR of higher acuity arrivals (ESI 1 and ESI 2) and a small numeric decrease in RR of lower-acuity presentations (ESI 3 and ESI 4 & 5), but these effects did not reach statistical significance (Figure 3, Table 2). At extreme temperatures, confidence intervals widened due to the small number of extremely hot or cold days in the dataset, limiting statistical significance. Similarly, for extremes of acuity (ESI 1 and ESI 4 & 5), statistical significance may have been constrained by smaller sample sizes.

## DISCUSSION

Our objective in this study was to analyze the relationship between temperature and ED arrivals across strata of patient acuity in a large, urban, academic medical center. To the best of our knowledge, the few studies examining the relationship between temperature and ED acuity have relied on predictive models, without specifically quantifying this relationship while accounting for lags and non-linear patterns.^10^–^13^ Here, we address this gap by employing a distributed lag non-linear Poisson regression to account for seasonality and the delayed health effects of heat across each acuity strata.

Total ED arrivals (aggregated across strata of acuity) increased with temperature, consistent with findings from other studies using EHR or claims data.^8^,^21^–^23^ When stratified by acuity, DLNMs revealed that most of the temperature-responsiveness of ED arrivals was in medium- to high-acuity cases (ESI 2 and ESI 3). Arrivals in these groups increased with temperature, even at temperatures well below the threshold typically associated with heat-related illness (HRI). During the hottest 5% of days, our data suggest that RR for ESI 2 arrivals continues to increase, whereas for ESI 3, the RR returns toward baseline. The highest acuity (ESI 1) and lowest acuity (ESI 4 & 5) cases did not demonstrate statistically significant associations with temperature. However, on the hottest 5% of days, ESI 1 arrivals exhibited a subtle numeric increase in RR of arrivals with increasing temperature, while ESI 4 and 5 displayed a subtle numeric decrease in RR of arrivals on the hottest days.

At this temperate-zone site, most heat-related illness cases are expected to occur within the upper 10% of temperatures. It is plausible that very hot weather could influence decisions to seek or not seek care for low-acuity conditions. While caution is warranted when interpreting these edge cases, the opposing directions in the upper tails of the DLNM curves at the highest temperatures—an upward numeric change for higher acuity cases and downward numeric change for lower acuity cases—are suggestive of different dynamics affecting heat-sensitive pathologies and/or care-seeking behavior between high- and low-ESI groups. These findings warrant further investigation using larger datasets and/or at sites with higher levels of heat exposure.

With regard to the lagged impact of temperature, we found the overall strongest effect on current day of exposure (lag 0), which aligns with current literature,^24^–^26^ although some heterogeneity existed between different ESI groups.

While additional studies, particularly in regions experiencing a higher frequency of extreme temperature days, will help clarify the relationship between temperature and the acuity of ED arrivals, the numeric effects noted in this study are worth examining. For instance, heat may increase the risk of decompensation of chronic conditions in addition to leading to cases of heatstroke or heat exhaustion, driving higher numeric RR values of high-acuity presentations in the hottest weather. This hypothesis aligns with existing literature that describes extreme heat as a driver for higher morbidity of cardiovascular, respiratory, renal, and psychiatric pathologies, which frequently lead to higher acuity arrivals.^4^–^6^,^9^ Conversely, for lower acuity presentations, adverse weather conditions may deter care-seeking behaviors, contributing to lower numeric RR of presentation. Interestingly, prior epidemiologic studies exploring the mortality-temperature relation have suggested a U- or J-shaped exposure-response curve,^19^, ^27^ where both high and low temperatures are associated with increased mortality, with greater effects observed at higher temperatures. Here, however, we found decreased risk of ED arrivals at lower temperatures, even in sub-zero winter weathera.[Table t1-wjem-26-1338]

## LIMITATIONS

While the large sample size and extended study period are important strengths of this analysis, some limitations must be considered. First, the data are from a single urban center located in a geographical region with a limited number of extreme heat days. This limits the generalizability of our findings to other geographical regions with different characteristics, including differences in physiologic, cultural, and infrastructure-related resilience to the effects of temperature extremes. Second, as noted above, the small number of days at the extreme ends of the temperature spectrum limited the power of our analysis in detecting statistically significant trends at the extremes, particularly for extreme heat, which is of growing interest in the setting of climate change. Additionally, the 95th percentile wet-bulb temperature in coastal Massachusetts observed during the study period likely poses less heat stress compared to temperatures at other locations in the United States, such as the Southern and Southwestern states. This may have further limited the observed impact of the hottest days on acuity of ED arrival, which would be expected to be more pronounced in regions with greater heat extremes.

Finally, ED arrivals were not stratified by traumatic vs non-traumatic etiologies or by specific diagnosis, which likely respond differently to temperature variations. Here, we focused on the acuity level upon arrival to the ED, while differences in management, length of stay, or admission rates were beyond the scope of this study. Future studies in diverse geographic regions, incorporating more detailed stratification of presenting etiologies, are warranted to address these limitations and enhance the power and generalizability of these findings.

Understanding the impact of extreme heat on ED arrivals and healthcare use is crucial for development of data-informed climate change adaptation and preparedness strategies. This study and future research examining how heat affects ED acuity may help administrators optimize staffing and resource allocation leading up to or during heat waves. Further stratification by clinical condition could provide valuable insight to inform targeted patient outreach and education programs, enabling healthcare systems to support patients in remaining safe during extreme weather events.

## CONCLUSION

In this retrospective study focusing on a temperate region, we found that extremes of temperature, particularly heat, appear to affect ED arrivals differently across different acuity levels. Hotter weather was associated with increased relative risk of moderate-to-high acuity ED arrivals, but relative risks of very high and low acuity arrivals were less associated with extremes of temperature. A non-statistically significant numerical increase in ESI 1 presentations, and a non-statistically significant numerical decrease in ESI 4 and ESI 5 presentations were noted on the hottest days.

This study did not directly evaluate operational costs, staffing impacts, or patient outcomes associated with these changes. To understand the potential impact of extreme heat on ED operations nationwide and inform data-driven climate change preparedness strategies, additional research is necessary on both arrival volumes and on these operationally relevant endpoints in climatically diverse geographic regions that may experience more frequent temperature extremes, ideally incorporating more detailed stratification of presenting etiologies.

## Supplementary Information



## Figures and Tables

**Table 1 t1-wjem-26-1338:** Summary characteristics of variables used in the analysis, including the percentage of days with data for each variable (eg, only 6.3 % of days had snowfall data). Median and range are reported for continuous variables.

Variable	Category	Units	Proportion of Dates
Date	index	-	[1/1/2010 – 12/30/2019]
Arrival count	outcome	arrivals per day	100% 152 [61 – 228]
ESI level	ordinal	rank of ESI, (ie, 1–5)	100%
Day of week	calendar	categorical day of the week (eg, Monday)	100%
Day of holiday	calendar	holiday, yes or no	3%
Day before holiday	calendar	day following a holiday, yes or no	3%
Day after holiday	calendar	day following a holiday, yes or no	3%
Precipitation	weather	mm of precipitation, daily total	35.7% 0.33 [0.01 – 3.40]
Snow	weather	mm of snow, daily total	6.3% 2.3 [0.1 – 22.1]
Wind speed	weather	average wind speed, meters/second	100% 10.8 [1.7 – 36.7]
Temperature max	weather	maximum daily wet-bulb temperature, °C	100% 10.8 [−13.9 – 27.2]

*ESI*, Emergency Severity Index; *mm*, millimeter.

**Table 2:** Relative risk of emergency department arrivals and the corresponding 95% confidence interval for each Emergency Severity Index category at lag 0 associated with various temperature points. Blue boxes indicate a statistically significant relative risk < 1, while orqange boxes indicate a statistically significant relative risk > 1.

Exposure temp	RR [CI] Total Arrivals	RR [CI] ESI 1	RR [CI] ESI 2	RR [CI] ESI 3	RR [CI] ESI 4 & 5
−13.89	0.94 [0.88 – 1.01]	0.95 [0.73 – 1.23]	0.96 [0.84 – 1.09]	0.93 [0.84 – 1.02]	1.00 [0.77 – 1.31]
−11.73	0.94 [0.90 – 0.98]	0.95 [0.82 – 1.11]	0.94 [0.87 – 1.01]	0.92 [0.87 – 0.98]	1.00 [0.86 – 1.17]
−9.56	0.94 [0.91 – 0.96]	0.96 [0.87 – 1.05]	0.93 [0.89 – 0.98]	0.93 [0.90 – 0.96]	1.00 [0.91 – 1.10]
−7.40	0.94 [0.92 – 0.96]	0.96 [0.89 – 1.04]	0.93 [0.90 – 0.97]	0.93 [0.91 – 0.96]	1.00 [0.93 – 1.08]
−5.23	0.95 [0.93 – 0.97]	0.96 [0.90 – 1.03]	0.94 [0.91 – 0.97]	0.94 [0.92 – 0.97]	1.00 [0.93 – 1.07]
−3.07	0.96 [0.94 – 0.97]	0.96 [0.91 – 1.02]	0.95 [0.93 – 0.98]	0.95 [0.93 – 0.97]	0.99 [0.94 – 1.05]
−0.91	0.97 [0.95 – 0.98]	0.96 [0.91 – 1.02]	0.96 [0.94 – 0.99]	0.96 [0.95 – 0.98]	0.99 [0.94 – 1.04]
1.26	0.97 [0.96 – 0.99]	0.97 [0.92 – 1.01]	0.97 [0.95 – 0.99]	0.97 [0.96 – 0.99]	0.99 [0.95 – 1.04]
3.42	0.98 [0.97 – 0.99]	0.97 [0.93 – 1.01]	0.98 [0.96 – 0.99]	0.98 [0.96 – 0.99]	0.99 [0.95 – 1.03]
5.58	0.98 [0.98 – 0.99]	0.98 [0.95 – 1.01]	0.99 [0.97 – 1.00]	0.98 [0.97 – 0.99]	0.99 [0.96 – 1.02]
7.75	0.99 [0.98 – 0.99]	0.98 [0.96 – 1.00]	0.99 [0.98 – 1.00]	0.99 [0.98 – 0.99]	0.99 [0.97 – 1.01]
9.91	0.99 [0.99 – 0.99]	0.99 [0.98 – 1.00]	0.99 [0.99 – 0.99]	0.99 [0.99 – 0.99]	1.00 [0.99 – 1.00]
12.08	1.01 [1.01 – 1.01]	1.00 [1.00 – 1.00]	1.01 [1.01 – 1.01]	1.01 [1.01 – 1.01]	1.00 [1.00 – 1.00]
14.24	1.01 [1.01 – 1.01]	1.01 [1.01 – 1.03]	1.01 [1.01 – 1.01]	1.01 [1.01 – 1.01]	1.01 [1.00 – 1.02]
16.40	1.02 [1.01 – 1.02]	1.02 [1.01 – 1.05]	1.02 [1.01 – 1.03]	1.01 [1.01 – 1.02]	1.01 [0.99 – 1.04]
18.57	1.03 [1.02 – 1.04]	1.04 [1.00 – 1.08]	1.03 [1.01 – 1.05]	1.02 [1.01 – 1.04]	1.02 [0.99 – 1.06]
20.73	1.04 [1.02 – 1.05]	1.05 [0.99 – 1.11]	1.04 [1.02 – 1.07]	1.04 [1.02 – 1.06]	1.03 [0.98 – 1.08]
22.89	1.04 [1.03 – 1.06]	1.04 [0.98 – 1.10]	1.04 [1.01 – 1.07]	1.05 [1.03 – 1.07]	1.03 [0.97 – 1.09]
25.06	1.04 [1.02 – 1.06]	1.02 [0.94 – 1.11]	1.06 [1.02 – 1.10]	1.04 [1.01 – 1.07]	0.98 [0.91 – 1.06]
27.22	1.02 [0.95 – 1.10]	1.06 [0.79 – 1.42]	1.16 [1.01 – 1.32]	0.97 [0.87 – 1.08]	0.83 [0.63 – 1.10]

Note: The relative risk of ED arrivals stratified by acuity for lag 0, lag 1, lag 2, lag 3, and a lag aggregate can be found in the Appendix.

*ESI*, Emergency Severity Index; *CI*, confidence interval; *RR*, relative risk.

## References

[1] Rohde R (2025). Global Temperature Report for 2024. Berkeley Earth.

[2] Intergovernmental Panel on Climate Change (IPCC) (2023). Climate Change 2023: Synthesis Report. Contribution of Working Groups I, II, and III to the Sixth Assessment Report of the Intergovernmental Panel on Climate Change. https://www.ipcc.ch/report/ar6/syr/.

[3] Dahl K, Licker R, Abatzoglou JT (2019). Increased frequency of and population exposure to extreme heat index days in the United States during the 21st century. Environ Res Commun.

[4] Liu J, Varghese BM, Hansen A (2022). Heat exposure and cardiovascular health outcomes: a systematic review and meta-analysis. Lancet Planet Health.

[5] Nori-Sarma A, Sun S, Sun Y (2022). Association between ambient heat and risk of emergency department visits for mental health among US Adults, 2010 to 2019. JAMA Psychiatry.

[6] im Kampe EO, Kovats S, Hajat S (2016). Impact of high ambient temperature on unintentional injuries in high-income countries: a narrative systematic literature review. BMJ Open.

[7] Nyadanu SD, Dunne J, Tessema GA (2024). Maternal exposure to ambient air temperature and adverse birth outcomes: an umbrella review of systematic reviews and meta-analyses. Sci Total Environ.

[8] Sun S, Weinberger KR, Nori-Sarma A (2021). Ambient heat and risks of emergency department visits among adults in the United States: time stratified case crossover study. BMJ.

[9] Basu R, Pearson D, Malig B (2012). The effect of high ambient temperature on emergency room visits. Epidemiology.

[10] Jiang S, Chin KS, Tsui KL (2018). A universal deep learning approach for modeling the flow of patients under different severities. Comput Methods Programs Biomed.

[11] Xu M, Wong TC, Chin KS (2011). Modeling patient visits to accident and emergency department in Hong Kong. IEEE, GBR.

[12] Sun Y, Heng BH, Seow YT (2009). Forecasting daily attendances at an emergency department to aid resource planning. BMC Emerg Med.

[13] Tai CC, Lee CC, Shih CL (2007). Effects of ambient temperature on volume, specialty composition and triage levels of emergency department visits. Emerg Med J.

[14] Peel MC, Finlayson BL, McMahon TA (2007). Updated world map of the Köppen-Geiger climate classification, Hydrol. Earth Syst Sci.

[15] Emergency Nurses Association (2023). Emergency Severity Index Handbook.

[16] Spangler KR, Adams QH, Hu JK (2023). Does choice of outdoor heat metric affect heat-related epidemiologic analyses in the US Medicare population?. Environ Epidemiol.

[17] Jiang S, Liu Q, Ding B (2023). A systematic review of the modelling of patient arrivals in emergency departments. Quant Imaging Med Surg.

[18] Gasparrini A (2014). Modeling exposure-lag-response associations with distributed lag non-linear models. Stat Med.

[19] Gasparrini A, Guo Y, Hashizume M (2015). Mortality risk attributable to high and low ambient temperature: a multicountry observational study. Lancet.

[20] Bao J, Wang Z, Yu C (2016). The influence of temperature on mortality and its lag effect: a study in four Chinese cities with different latitudes. BMC Public Health.

[21] Zhao Q, Zhang Y, Zhang W (2017). Ambient temperature and emergency department visits: time-series analysis in 12 Chinese cities. Environ Pollut.

[22] Knowlton K, Rotkin-Ellman M, King G (2009). The 2006 California heat wave: impacts on hospitalizations and emergency department visits. Environ Health Perspect.

[23] Bernstein AS, Sun S, Weinberger KR (2022). Warm season and emergency department visits to U.S. children’s hospitals. Environ Health Perspect.

[24] Rocklöv J, Forsberg B (2008). The effect of temperature on mortality in Stockholm 1998–2003: a study of lag structures and heatwave effects. Scand J Public Health.

[25] Lian T, Fu Y, Sun M (2020). Effect of temperature on accidental human mortality: a time-series analysis in Shenzhen, Guangdong Province in China. Sci Rep.

[26] Yang J, Ou CQ, Ding Y (2012). Daily temperature and mortality: a study of distributed lag non-linear effect and effect modification in Guangzhou. Environ Health.

[27] Curriero FC, Heiner KS, Samet JM (2002). Temperature and mortality in 11 cities of the Eastern United States. Am J Epidemiol.

