# Supplementary material for: Impact of Daily Maximum Temperature on Emergency Department Arrivals and Acuity Levels
Source: West J Emerg Med. 2025 Sep 25;26(5):1338–44. doi: 10.5811/westjem.42263 (PMC12591613; doi:10.5811/westjem.42263)
Supplement: Supplementary file 1 [file wjem-26-1338-s001.docx]

**SUPPLEMENTARY APPENDIX**

**Daily Model Selection**

Model selection was based on literature review. Sensitivity analysis was conducted to test the robustness of changing model choices for daily lagged models. We used Akaike information criteria (AIC) values to help guide model selection.

**SM Table 1.** AIC values for models using different temperature variables. In our model we used wet-bulb maximum as it combines both ambient temperature and humidity and may better correlate with physiological response to heat. The black outline represents the temperature variable used in the model.

|  | **Wet-Bulb Min** | **Wet-Bulb Avg** | **Wet-Bulb Max** | **Dry-Bulb Min** | **Dry-Bulb Avg** | **Dry-Bulb Max** |
| --- | --- | --- | --- | --- | --- | --- |
| **Total Arrivals** | 29925.43 | 29909.26 | 29914.93 | 29932.89 | 29909.58 | 29915.65 |
| **ESI1** | 21646.48 | 21643.89 | 21636.7 | 21635.99 | 21638.42 | 21642.49 |
| **ESI2** | 27989.79 | 28002.23 | 28015.09 | 28004.07 | 28008.82 | 28614.35 |
| **ESI3** | 28596.63 | 28607.53 | 28619.13 | 28604.6 | 28610.05 | 28614.35 |
| **ESI4+5** | 23481.06 | 23495.47 | 23523.22 | 23471.46 | 23487.21 | 23506.02 |

**SM Table 2.** AIC values [CD3] [MOU4] for models with differing lag periods for weather variables. Final lag decisions were based predominantly on literature analysis. The black outline represents the lag value used in model for each weather variable.

**Wet-Bulb Temperature, Maximum**

| **Lag value** | **1** | **2** | **3** | **4** | **5** |
| --- | --- | --- | --- | --- | --- |
| **Total** | 29923.65 | 29911.89 | 29916.66 | 29915.47 | 29908.26 |
| **ESI1** | 21630.8 | 21631.45 | 21631.01 | 21625.69 | 21614.25 |
| **ESI2** | 28032.47 | 28025.88 | 28024.49 | 28024.93 | 28013.84 |
| **ESI3** | 28613.51 | 28608.9 | 28616.05 | 28613.41 | 28604.67 |
| **ESI4+5** | 23553.57 | 23547.4 | 23538.45 | 23499.25 | 23482.53 |

**Precipitation, Daily Total**

| **Lag value** | **1** | **2** | **3** | **4** | **5** | **14** | **21** |
| --- | --- | --- | --- | --- | --- | --- | --- |
| **Total** | 29906.99 | 29911.35 | 29916.66 | 29908.08 | 29902.59 | 29830 | 29779.32 |
| **ESI1** | 21622.1 | 21623.95 | 21631.01 | 21621.76 | 21611.26 | 21485.36 | 21398.7 |
| **ESI2** | 28014.1 | 28017.56 | 28024.49 | 28015.6 | 27976.54 | 27852.96 | 27774.6 |
| **ESI3** | 28610.56 | 28610.75 | 28616.05 | 28613.04 | 28588.13 | 28508.79 | 28437.93 |
| **ESI4+5** | 23551.57 | 23548.64 | 23538.45 | 23537.62 | 23517.13 | 23409.53 | 23354.78 |

**Snow, Daily Total**

| **Lag value** | **1** | **2** | **3** | **4** | **5** | **14** | **21** |
| --- | --- | --- | --- | --- | --- | --- | --- |
| **Total** | 29918.37 | 29908.54 | 29908.17 | 29888.6 | 29884.7 | 29943.05 | 29951.82 |
| **ESI1** | 21627.81 | 21628.17 | 21630.92 | 21622.33 | 21620.6 | 21566.76 | 21519.31 |
| **ESI2** | 28018.68 | 28010.64 | 28012.71 | 28009.05 | 27999.7 | 27986.66 | 27954.66 |
| **ESI3** | 28601.63 | 28604.83 | 28608.29 | 28590.38 | 28590.17 | 28565.95 | 28518.14 |
| **ESI4+5** | 23554.32 | 23538.14 | 23534.27 | 23540.46 | 23535.39 | 23488.47 | 23428.7 |

**Wind Speed, Average**

| **Lag value** | **1** | **2** | **3** | **4** | **5** |
| --- | --- | --- | --- | --- | --- |
| **Total** | 29914.93 | 29924.91 | 29934.22 | 29920.87 | 29912.92 |
| **ESI1** | 21636.7 | 21638.17 | 21626.45 | 21619.81 | 21598.48 |
| **ESI2** | 28015.09 | 27996.65 | 27989.37 | 27970.72 | 27943.06 |
| **ESI3** | 28619.13 | 28628.8 | 28636.44 | 28620.46 | 28608.46 |
| **ESI4+5** | 23523.22 | 23525.17 | 23525.94 | 23511.52 | 23496.88 |

**SM Table 3.** Relative risk of total ED arrival by exposure temperature for various lags. Blue boxes indicate a statistically significant relative risk less than 1, while red boxes indicate a statistically significant relative risk greater than 1.

| **Exposure temp** | **RR [CI] All Lags** | **RR [CI] Lag 0** | **RR [CI] Lag 1** | **RR [CI] Lag 2** | **RR [CI] Lag 3** |
| --- | --- | --- | --- | --- | --- |
| -13.89 | 0.80 [0.52 - 1.24] | 0.94 [0.88 - 1.01] | 0.93 [0.87 - 1.01] | 1.07 [1.00 - 1.15] | 1.02 [0.95 - 1.09] |
| -11.73 | 0.76 [0.61 - 0.95] | 0.94 [0.90 - 0.98] | 0.96 [0.91 – 0.99] | 1.03 [0.98 - 1.07] | 1.00 [0.96 - 1.04] |
| -9.56 | 0.75 [0.67 - 0.85] | 0.94 [0.91 - 0.96] | 0.97 [0.94 – 0.99] | 1.00 [0.97 - 1.03] | 0.99 [0.96 - 1.01] |
| -7.40 | 0.77 [0.70 - 0.84] | 0.94 [0.92 - 0.96] | 0.98 [0.96 - 1.01] | 0.99 [0.97 - 1.02] | 0.98 [0.96 - 1.00] |
| -5.23 | 0.80 [0.74 - 0.88] | 0.95 [0.93 - 0.97] | 0.99 [0.97 - 1.01] | 1.00 [0.98 - 1.02] | 0.98 [0.96 - 0.99] |
| -3.07 | 0.85 [0.79 - 0.91] | 0.96 [0.94 - 0.97] | 0.99 [0.97 - 1.01] | 1.01 [0.99 - 1.02] | 0.98 [0.97 - 0.99] |
| -0.91 | 0.89 [0.84 - 0.95] | 0.97 [0.95 - 0.98] | 0.99 [0.98 - 1.01] | 1.01 [1.00 - 1.03] | 0.98 [0.97 - 0.99] |
| 1.26 | 0.93 [0.87 - 0.98] | 0.97 [0.96 - 0.99] | 0.99 [0.98 - 1.01] | 1.02 [1.01 - 1.03] | 0.99 [0.98 - 0.99] |
| 3.42 | 0.95 [0.90 - 0.99] | 0.98 [0.97 - 0.99] | 1.00 [0.98 - 1.01] | 1.02 [1.01 - 1.03] | 0.99 [0.98 - 1.00] |
| 5.58 | 0.97 [0.93 - 1.00] | 0.98 [0.98 - 0.99] | 1.00 [0.99 - 1.01] | 1.01 [1.01 - 1.02] | 0.99 [0.99 - 1.00] |
| 7.75 | 0.98 [0.95 - 1.00] | 0.99 [0.98 - 0.99] | 1.00 [0.99 - 1.00] | 1.01 [1.01 - 1.02] | 1.00 [0.99 - 1.00] |
| 9.91 | 0.99 [0.98 - 1.00] | 0.99 [0.99 - 0.99] | 1.00 [1.00 - 1.00] | 1.01 [1.01 - 1.01] | 1.00 [1.00 - 1.00] |
| 12.08 | 1.01 [1.01 - 1.01] | 1.01 [1.01 - 1.01] | 1.00 [1.00 - 1.00] | 0.99 [0.99 - 0.99] | 1.00 [1.00 - 1.00] |
| 14.24 | 1.02 [1.01 - 1.04] | 1.01 [1.01 - 1.01] | 1.00 [1.00 - 1.00] | 0.99 [0.99 -0.99] | 1.00 [1.00 - 1.01] |
| 16.40 | 1.05 [1.03 - 1.08] | 1.02 [1.01 - 1.02] | 1.00 [0.99 - 1.01] | 1.00 [0.99 - 1.00] | 1.00 [1.00 - 1.01] |
| 18.57 | 1.10 [1.05 - 1.15] | 1.03 [1.02 - 1.04] | 1.00 [0.99 - 1.01] | 1.00 [0.99 - 1.01] | 1.00 [0.99 - 1.01] |
| 20.73 | 1.16 [1.09 - 1.24] | 1.04 [1.02 - 1.05] | 1.00 [0.99 - 1.02] | 1.01 [0.99 - 1.02] | 1.00 [0.99 - 1.02] |
| 22.89 | 1.23 [1.15 - 1.31] | 1.04 [1.03 - 1.06] | 1.00 [0.98 - 1.02] | 1.02 [1.00 - 1.04] | 1.01 [0.99 - 1.02] |
| 25.06 | 1.15 [1.06 - 1.25] | 1.04 [1.02 - 1.06] | 0.99 [0.96 - 1.01] | 1.01 [0.99 - 1.04] | 1.01 [0.99 - 1.03] |
| 27.22 | 0.76 [0.49 - 1.18] | 1.02 [0.95 - 1.10] | 0.97 [0.90 - 1.05] | 0.97 [0.89 - 1.05] | 1.01 [0.94 - 1.09] |
|  |  |  |  |  |  |

**SM Table 4.** Relative risk of ESI 1 arrival by exposure temperature for various lags. Blue boxes indicate a statistically significant relative risk less than 1, while red boxes indicate a statistically significant relative risk greater than 1.

| **Exposure temp** | **RR [CI] All Lags** | **RR [CI] Lag 0** | **RR [CI] Lag 1** | **RR [CI] Lag 2** | **RR [CI] Lag 3** |
| --- | --- | --- | --- | --- | --- |
| -13.89 | 0.77 [0.51 - 1.18] | 0.95 [0.73 - 1.23] | 0.81 [0.61 - 1.08] | 1.05 [0.81 - 1.37] | 0.95 [0.73 - 1.25] |
| -11.73 | 0.90 [0.72 - 1.11] | 0.95 [0.82 - 1.11] | 0.96 [0.81 - 1.14] | 0.99 [0.84 - 1.15] | 0.99 [0.85 - 1.15] |
| -9.56 | 0.98 [0.87 - 1.10] | 0.96 [0.87 - 1.05] | 1.06 [0.95 - 1.18] | 0.95 [0.86 - 1.06] | 1.01 [0.92 - 1.11] |
| -7.40 | 1.01 [0.93 - 1.11] | 0.96 [0.89 - 1.04] | 1.10 [1.01 - 1.20] | 0.95 [0.87 - 1.04] | 1.01 [0.94 - 1.09] |
| -5.23 | 1.02 [0.94 - 1.11] | 0.96 [0.90 - 1.03] | 1.10 [1.02 - 1.19] | 0.96 [0.89 - 1.04] | 1.01 [0.94 - 1.07] |
| -3.07 | 1.01 [0.94 - 1.08] | 0.96 [0.91 - 1.02] | 1.08 [1.01 - 1.15] | 0.98 [0.91 - 1.04] | 0.99 [0.94 - 1.05] |
| -0.91 | 0.99 [0.93 - 1.06] | 0.96 [0.91 - 1.02] | 1.05 [0.99 - 1.11] | 1.00 [0.94 - 1.06] | 0.98 [0.94 - 1.04] |
| 1.26 | 0.98 [0.92 - 1.04] | 0.97 [0.92 - 1.01] | 1.02 [0.97 - 1.08] | 1.01 [0.96 - 1.06] | 0.98 [0.94 - 1.03] |
| 3.42 | 0.98 [0.93 - 1.03] | 0.97 [0.93 - 1.01] | 1.01 [0.97 - 1.06] | 1.02 [0.97 - 1.06] | 0.98 [0.95 - 1.02] |
| 5.58 | 0.98 [0.95 - 1.02] | 0.98 [0.95 - 1.01] | 1.00 [0.97 - 1.04] | 1.02 [0.98 - 1.05] | 0.99 [0.96 - 1.02] |
| 7.75 | 0.99 [0.96 - 1.02] | 0.98 [0.96 - 1.00] | 1.00 [0.98 - 1.02] | 1.01 [0.99 - 1.04] | 0.99 [0.97 - 1.01] |
| 9.91 | 1.00 [0.99 - 1.01] | 0.99 [0.98 - 1.00] | 1.00 [0.99 - 1.01] | 1.01 [1.00 - 1.02] | 1.00 [0.99 - 1.01] |
| 12.08 | 1.00 [1.00 - 1.00] | 1.00 [1.00 - 1.00] | 1.00 [1.00 - 1.00] | 1.00 [1.00 - 1.00] | 1.00 [1.00 - 1.00] |
| 14.24 | 1.00 [0.98 - 1.01] | 1.01 [1.01 - 1.03] | 1.00 [0.99 - 1.01] | 0.99 [0.98 - 1.00] | 1.00 [0.98 - 1.01] |
| 16.40 | 0.99 [0.96 - 1.02] | 1.02 [1.01 - 1.05] | 1.00 [0.97 - 1.02] | 0.98 [0.95 - 1.01] | 0.99 [0.96 - 1.01] |
| 18.57 | 0.97 [0.92 - 1.02] | 1.04 [1.00 - 1.08] | 0.99 [0.95 - 1.03] | 0.97 [0.93 - 1.02] | 0.97 [0.93 - 1.00] |
| 20.73 | 0.95 [0.89 - 1.02] | 1.05 [0.99 - 1.11] | 0.98 [0.92 - 1.05] | 0.97 [0.92 - 1.04] | 0.95 [0.90 - 0.99] |
| 22.89 | 1.00 [0.93 - 1.07] | 1.04 [0.98 - 1.10] | 1.00 [0.93 - 1.08] | 1.00 [0.93 - 1.07] | 0.96 [0.90 - 1.02] |
| 25.06 | 0.90 [0.82 - 0.99] | 1.02 [0.94 - 1.11] | 0.98 [0.89 - 1.08] | 0.99 [0.90 - 1.09] | 0.91 [0.84 - 0.99] |
| 27.22 | 0.41 [0.25 - 0.67] | 1.06 [0.79 - 1.42] | 0.76 [0.55 - 1.05] | 0.82 [0.60 - 1.13] | 0.62 [0.46 - 0.86] |
|  |  |  |  |  |  |

**SM Table 5.** Relative risk of ESI 2 arrival by exposure temperature for various lags. Blue boxes indicate a statistically significant relative risk less than 1, while red boxes indicate a statistically significant relative risk greater than 1.

| **Exposure temp** | **RR [CI] All Lags** | **RR [CI] Lag 0** | **RR [CI] Lag 1** | **RR [CI] Lag 2** | **RR [CI] Lag 3** |
| --- | --- | --- | --- | --- | --- |
| -13.89 | 1.13 [0.92 - 1.37] | 0.96 [0.84 - 1.09] | 1.04 [0.92 - 1.18] | 1.05 [0.92 - 1.19] | 1.07 [0.95 - 1.21] |
| -11.73 | 1.01 [0.91 - 1.11] | 0.94 [0.87 - 1.01] | 1.00 [0.92 - 1.08] | 1.03 [0.96 - 1.11] | 1.04 [0.97 - 1.11] |
| -9.56 | 0.95 [0.90 - 1.00] | 0.93 [0.89 - 0.98] | 0.97 [0.92 - 1.02] | 1.03 [0.98 - 1.08] | 1.02 [0.97 - 1.06] |
| -7.40 | 0.93 [0.89 - 0.97] | 0.93 [0.90 - 0.97] | 0.96 [0.92 - 1.01] | 1.03 [0.99 - 1.07] | 1.00 [0.97 - 1.04] |
| -5.23 | 0.94 [0.90 - 0.98] | 0.94 [0.91 - 0.97] | 0.97 [0.93 - 1.00] | 1.04 [1.00 - 1.07] | 1.00 [0.97 - 1.03] |
| -3.07 | 0.96 [0.93 - 0.99] | 0.95 [0.93 - 0.98] | 0.97 [0.94 - 1.00] | 1.04 [1.01 - 1.08] | 1.00 [0.97 - 1.02] |
| -0.91 | 0.99 [0.96 - 1.02] | 0.96 [0.94 - 0.99] | 0.98 [0.96 - 1.01] | 1.05 [1.02 - 1.07] | 1.00 [0.98 - 1.02] |
| 1.26 | 1.01 [0.98 - 1.03] | 0.97 [0.95 - 0.99] | 0.99 [0.97 - 1.01] | 1.04 [1.02 - 1.07] | 1.00 [0.98 - 1.02] |
| 3.42 | 1.01 [0.99 - 1.04] | 0.98 [0.96 – 0.99] | 0.99 [0.97 - 1.02] | 1.04 [1.02 - 1.06] | 1.00 [0.98 - 1.02] |
| 5.58 | 1.01 [0.99 - 1.03] | 0.99 [0.97 - 1.00] | 1.00 [0.98 - 1.01] | 1.03 [1.01 - 1.05] | 1.00 [0.99 - 1.01] |
| 7.75 | 1.01 [1.00 - 1.02] | 0.99 [0.98 - 1.00] | 1.00 [0.99 - 1.01] | 1.02 [1.01 - 1.03] | 1.00 [0.99 - 1.01] |
| 9.91 | 1.00 [1.00 - 1.01] | 0.99 [0.99 - 0.99] | 1.00 [1.00 - 1.00] | 1.01 [1.01 - 1.01] | 1.00 [1.00 - 1.00] |
| 12.08 | 1.00 [1.00 - 1.00] | 1.01 [1.01 - 1.01] | 1.00 [1.00 - 1.00] | 0.99 [0.99 - 0.99] | 1.00 [1.00 - 1.00] |
| 14.24 | 1.00 [0.99 - 1.01] | 1.01 [1.01 - 1.01] | 1.00 [0.99 - 1.01] | 0.99 [0.98 - 0.99] | 1.00 [1.00 - 1.01] |
| 16.40 | 1.00 [0.99 - 1.02] | 1.02 [1.01 - 1.03] | 1.00 [0.99 - 1.01] | 0.98 [0.97 - 0.99] | 1.00 [0.99 - 1.01] |
| 18.57 | 1.02 [1.00 - 1.04] | 1.03 [1.01 - 1.05] | 1.00 [0.98 - 1.02] | 0.98 [0.96 - 1.00] | 1.01 [0.99 - 1.02] |
| 20.73 | 1.03 [1.00 - 1.07] | 1.04 [1.02 - 1.07] | 1.00 [0.97 - 1.03] | 0.99 [0.96 - 1.02] | 1.01 [0.98 - 1.03] |
| 22.89 | 1.00 [0.97 - 1.04] | 1.04 [1.01 - 1.07] | 0.98 [0.94 - 1.01] | 1.00 [0.96 - 1.03] | 0.99 [0.96 - 1.02] |
| 25.06 | 1.03 [0.99 - 1.07] | 1.06 [1.02 - 1.10] | 0.97 [0.93 - 1.01] | 0.99 [0.95 - 1.04] | 1.01 [0.98 - 1.05] |
| 27.22 | 1.44 [1.17 - 1.77] | 1.16 [1.01 - 1.32] | 1.07 [0.93 - 1.23] | 0.94 [0.82 - 1.08] | 1.24 [1.09 - 1.41] |
|  |  |  |  |  |  |

**SM Table 6.** Relative risk of ESI 3 arrival by exposure temperature for various lags. Blue boxes indicate a statistically significant relative risk less than 1, while red boxes indicate a statistically significant relative risk greater than 1.

| **Exposure temp** | **RR [CI] All Lags** | **RR [CI] Lag 0** | **RR [CI] Lag 1** | **RR [CI] Lag 2** | **RR [CI] Lag 3** |
| --- | --- | --- | --- | --- | --- |
| -13.89 | 0.92 [0.79 - 1.08] | 0.93 [0.84 - 1.02] | 0.91 [0.82 - 1.01] | 1.10 [0.99 - 1.21] | 1.00 [0.91 - 1.10] |
| -11.73 | 0.89 [0.83 - 0.97] | 0.92 [0.87 - 0.98] | 0.95 [0.89 - 1.00] | 1.04 [0.98 - 1.10] | 0.99 [0.93 - 1.04] |
| -9.56 | 0.89 [0.85 - 0.92] | 0.93 [0.90 - 0.96] | 0.97 [0.93 - 1.01] | 1.00 [0.97 - 1.04] | 0.98 [0.95 - 1.01] |
| -7.40 | 0.89 [0.86 - 0.92] | 0.93 [0.91 - 0.96] | 0.99 [0.96 - 1.02] | 0.99 [0.96 - 1.02] | 0.98 [0.95 - 1.00] |
| -5.23 | 0.90 [0.88 - 0.93] | 0.94 [0.92 - 0.97] | 0.99 [0.97 - 1.02] | 0.99 [0.96 - 1.02] | 0.98 [0.95 - 1.00] |
| -3.07 | 0.92 [0.90 - 0.95] | 0.95 [0.93 - 0.97] | 1.00 [0.97 - 1.02] | 0.99 [0.97 - 1.02] | 0.98 [0.96 - 1.00] |
| -0.91 | 0.94 [0.92 - 0.97] | 0.96 [0.95 - 0.98] | 1.00 [0.97 - 1.02] | 1.00 [0.98 - 1.02] | 0.98 [0.97 - 1.00] |
| 1.26 | 0.96 [0.94 - 0.98] | 0.97 [0.96 - 0.99] | 1.00 [0.98 - 1.01] | 1.00 [0.98 - 1.02] | 0.99 [0.97 - 1.01] |
| 3.42 | 0.97 [0.95 - 0.99] | 0.98 [0.96 - 0.99] | 1.00 [0.98 - 1.01] | 1.00 [0.99 - 1.02] | 0.99 [0.98 - 1.01] |
| 5.58 | 0.98 [0.97 - 0.99] | 0.98 [0.97 - 0.99] | 1.00 [0.98 - 1.01] | 1.00 [0.99 - 1.02] | 1.00 [0.98 - 1.01] |
| 7.75 | 0.99 [0.98 - 0.99] | 0.99 [0.98 - 0.99] | 1.00 [0.99 - 1.01] | 1.00 [0.99 - 1.01] | 1.00 [0.99 - 1.00] |
| 9.91 | 0.99 [0.99 - 0.99] | 0.99 [0.99 - 0.99] | 1.00 [1.00 - 1.00] | 1.00 [1.00 - 1.00] | 1.00 [1.00 - 1.00] |
| 12.08 | 1.01 [1.01 - 1.01] | 1.01 [1.01 - 1.01] | 1.00 [1.00 - 1.00] | 1.00 [1.00 - 1.00] | 1.00 [1.00 - 1.00] |
| 14.24 | 1.01 [1.01 - 1.02] | 1.01 [1.01 - 1.01] | 1.00 [1.00 - 1.01] | 1.00 [1.00 - 1.01] | 1.00 [1.00 - 1.00] |
| 16.40 | 1.02 [1.01 - 1.03] | 1.01 [1.01 - 1.02] | 1.00 [0.99 - 1.01] | 1.00 [0.99 - 1.01] | 1.00 [0.99 - 1.01] |
| 18.57 | 1.04 [1.02 - 1.06] | 1.02 [1.01 - 1.04] | 1.00 [0.99 - 1.02] | 1.01 [1.00 - 1.03] | 1.00 [0.99 - 1.01] |
| 20.73 | 1.06 [1.03 - 1.09] | 1.04 [1.02 - 1.06] | 1.00 [0.98 - 1.03] | 1.02 [1.00 - 1.04] | 1.00 [0.98 - 1.02] |
| 22.89 | 1.09 [1.06 - 1.12] | 1.05 [1.03 - 1.07] | 1.00 [0.98 - 1.03] | 1.03 [1.01 - 1.06] | 1.01 [0.98 - 1.03] |
| 25.06 | 1.06 [1.03 - 1.10] | 1.04 [1.01 - 1.07] | 0.99 [0.95 - 1.02] | 1.03 [1.00 - 1.07] | 1.00 [0.97 - 1.03] |
| 27.22 | 0.87 [0.74 - 1.03] | 0.97 [0.87 - 1.08] | 0.94 [0.84 - 1.05] | 1.02 [0.91 - 1.14] | 0.94 [0.85 - 1.05] |
|  |  |  |  |  |  |

**SM Table 7.** Relative risk of ESI 4 & 5 arrival by exposure temperature for various lags. Blue boxes indicate a statistically significant relative risk less than 1, while red boxes indicate a statistically significant relative risk greater than 1.

| **Exposure temp** | **RR [CI] All Lags** | **RR [CI] Lag 0** | **RR [CI] Lag 1** | **RR [CI] Lag 2** | **RR [CI] Lag 3** |
| --- | --- | --- | --- | --- | --- |
| -13.89 | 0.80 [0.52 - 1.24] | 1.00 [0.77 - 1.31] | 0.79 [0.59 - 1.05] | 1.02 [0.77 - 1.34] | 1.00 [0.77 - 1.31] |
| -11.73 | 0.76 [0.61 - 0.95] | 1.00 [0.86 - 1.17] | 0.83 [0.70 - 0.99] | 0.95 [0.81 - 1.12] | 0.95 [0.82 - 1.11] |
| -9.56 | 0.75 [0.67 - 0.85] | 1.00 [0.91 - 1.10] | 0.87 [0.78 - 0.97] | 0.93 [0.83 - 1.03] | 0.93 [0.85 - 1.02] |
| -7.40 | 0.77 [0.70 - 0.84] | 1.00 [0.93 - 1.08] | 0.91 [0.83 - 0.99] | 0.93 [0.85 - 1.01] | 0.91 [0.85 - 0.98] |
| -5.23 | 0.80 [0.74 - 0.88] | 1.00 [0.93 - 1.07] | 0.94 [0.87 - 1.01] | 0.95 [0.88 - 1.02] | 0.91 [0.85 - 0.97] |
| -3.07 | 0.85 [0.79 - 0.91] | 0.99 [0.94 - 1.05] | 0.96 [0.90 - 1.02] | 0.98 [0.91 - 1.04] | 0.91 [0.86 - 0.97] |
| -0.91 | 0.89 [0.84 - 0.95] | 0.99 [0.94 - 1.04] | 0.98 [0.92 - 1.03] | 1.00 [0.95 - 1.06] | 0.92 [0.88 - 0.97] |
| 1.26 | 0.93 [0.87 - 0.98] | 0.99 [0.95 - 1.04] | 0.99 [0.94 - 1.04] | 1.02 [0.97 - 1.07] | 0.93 [0.89 - 0.97] |
| 3.42 | 0.95 [0.90 - 0.99] | 0.99 [0.95 - 1.03] | 0.99 [0.95 - 1.04] | 1.02 [0.98 - 1.07] | 0.94 [0.91 - 0.98] |
| 5.58 | 0.97 [0.93 - 1.00] | 0.99 [0.96 - 1.02] | 1.00 [0.96 - 1.03] | 1.02 [0.99 - 1.06] | 0.96 [0.93 - 0.98] |
| 7.75 | 0.98 [0.95 - 1.00] | 0.99 [0.97 - 1.01] | 1.00 [0.98 - 1.02] | 1.02 [0.99 - 1.04] | 0.97 [0.95 - 0.99] |
| 9.91 | 0.99 [0.98 - 1.00] | 1.00 [0.99 - 1.00] | 1.00 [0.99 - 1.01] | 1.01 [1.00 - 1.02] | 0.99 [0.98 - 0.99] |
| 12.08 | 1.00 [1.00 - 1.01] | 1.00 [1.00 - 1.00] | 1.00 [1.00 - 1.00] | 1.00 [1.00 - 1.00] | 1.01 [1.01 - 1.01] |
| 14.24 | 1.02 [1.01 - 1.04] | 1.01 [1.00 - 1.02] | 1.00 [0.99 - 1.02] | 0.99 [0.98 - 1.01] | 1.02 [1.01 - 1.03] |
| 16.40 | 1.05 [1.03 - 1.08] | 1.01 [0.99 - 1.04] | 1.01 [0.98 - 1.03] | 0.99 [0.97 - 1.02] | 1.04 [1.02 - 1.06] |
| 18.57 | 1.10 [1.05 - 1.15] | 1.02 [0.99 - 1.06] | 1.01 [0.97 - 1.06] | 1.00 [0.96 - 1.04] | 1.06 [1.02 - 1.10] |
| 20.73 | 1.16 [1.09 - 1.24] | 1.03 [0.98 - 1.08] | 1.03 [0.97 - 1.09] | 1.02 [0.96 - 1.08] | 1.08 [1.03 - 1.14] |
| 22.89 | 1.23 [1.15 - 1.31] | 1.03 [0.97 - 1.09] | 1.05 [0.98 - 1.12] | 1.02 [0.95 - 1.09] | 1.11 [1.05 - 1.18] |
| 25.06 | 1.15 [1.06 - 1.25] | 0.98 [0.91 - 1.06] | 1.07 [0.98 - 1.17] | 0.98 [0.90 - 1.07] | 1.11 [1.03 - 1.20] |
| 27.22 | 0.76 [0.49 - 1.18] | 0.83 [0.63 - 1.10] | 1.07 [0.81 - 1.41] | 0.88 [0.66 - 1.17] | 0.98 [0.74 - 1.29] |
|  |  |  |  |  |  |
